# Magnitude and determinants of stunting among children under five years of age in Ethiopia: a systematic review and meta-analysis

**DOI:** 10.3389/fped.2025.1499921

**Published:** 2025-06-09

**Authors:** Teshome Bekele Elema, Fetene Nega, Jemal Mohammed Ali, Gemechu Edae, Dube Jara Boneya

**Affiliations:** ^1^ Clinical Nutrition, Centre for Food Science and Nutrition, Addis Ababa University, Addis Ababa, Ethiopia; ^2^ Functional Foods and Food Fortification, College of Agriculture & Environmental Science, Arsi University, Asella, Ethiopia; ^3^ Assistant Professor of Food Science and Nutrition, College of Agriculture & Environmental Science, Arsi University, Asella, Ethiopia; ^4^ College of Health and Medical Science, Arsi University, Asella, Ethiopia; ^5^ Department of Public Health, Debre Markos University and Ph.D. Candidate in Public Health, School of Public Health College of Health Sciences, Addis Ababa University, Addis Ababa, Ethiopia

**Keywords:** stunting, underfive children, meta-analysis, PRISMA, systematic review, Ethiopia

## Abstract

**Background:**

There is regional variation in the incidence of stunting, the most common of which occurs in Amhara, the largest share of which is in Ethiopia. However, evidence on the magnitude of stunting and its determinants in children under five years of age is inadequate. The objective of the current review is to identify, appraise, and review systematically and to analyze the pooled effect of stunting among children under the age of five in Ethiopia.

**Materials and methods:**

The protocol of this review is registered in PROSPERO with registration number CRD42023323568 (https://www.crd.york.ac.uk). To combine the search results, we used reference management software (Endnote V-X7.2) and removed duplicates. For the meta-analysis, the Joanna Briggs Institute (JBI)-Meta-Analysis of Statistics Assessment and Review Instrument (MAStARI) was used for the critical appraisal of the studies. The data were categorized after extraction, sorted by quality score, and entered into STATA V14 for analysis. Cochrane's Q statistic (chi-square), *I*^2,^ and *p* values were used to check for heterogeneity in the studies' outcomes.

**Results:**

A total of 27 potentially eligible articles containing 32,448 under-five children were identified and included. The prevalence of stunting ranged from Eastern Hararghe (12.45%), the least prevalent to the highest prevalence, in the Tigray and Northwest regions (56.65%). Based on the meta-analysis, the overall pooled incidence of stunting was 40.30% (CI: 37.11–43.49 at 95% CI). The pooled effect size of twenty-seven studies showed that male children were 1.13 times more likely to have a risk of stunting than females (RR = 1.13, CI 1.01–1.26 @95%).

**Discussion and conclusions:**

Childhood stunting was significantly associated with the age of the child, child weight at birth, mother BMI, diarrhea episodes, deprivation of colostrum, and duration of breastfeeding. The issue of gender can be solved considering that breastfeeding duration was the lowest for daughters, as their parents were trying for a son. Finally, women's education is an alternative mechanism and sustainable strategy for overcoming the burden of childhood stunting.

**Systematic Review Registration:**

[http://www.library.UCSF.edu], identifier [CRD42023323568].

## Background

In Ethiopia, childhood stunting is a major public health concern, and the prevalence of childhood stunting has decreased over the past decade; however, the incidence of childhood stunting has remained high, with 38% of children under five stunted stunted and 18% being severely stunted (1). Evidence indicates that there is regional variation in the prevalence of stunting, in which the Amhara region accounts for the largest share (37%) of the population (1). According to the 2016 EDHS report, under 5 deaths were 67 per 1,000 live births. Stunting results from long-term inadequate food intake, poor-quality dietary access, a gradual increase in morbidity, or a combination of different factors (2). In Africa, the prevalence of chronic malnutrition has increased, and a WHO report indicated 40% stunting in the last decade (i.e., 2020) (3).

The significance of childhood stunting is comprehensive and includes both mortality and morbidity, low behavioral exploration, a high level of anxiety or depression, chronic diseases, poor health status, and a decrease in the intelligence quotient (IQ), which contributes to the cognitive performance of the child through their productive life (4, 5). However, there is a limitation to the power of statistical evidence on the level and determinant factors of stunting among children under five years of age. Several scholars have reported that parental influence and socioeconomic factors are considered determinants of stunting in developing countries. In a country such as Ethiopia, because of a lack of evidence-based intervention, improper management of data and a lack of skilled management contributed to significant effects among children under five stunted children (6). The above studies revealed inconsistency in the data across the different regions of Ethiopia.

For example, according to Berhe's discussion of seven factors that can be significantly associated with stunting, there was no significant interaction between the contributing variables (6). However, family size and low birth weight (LBW) contributed significantly to these findings. The incidence of undernutrition or stunting in children is high in Ethiopia, and considerable investment is needed in evidence-based nutritional interventions as well as research (7, 8). According to the above reports, the prevalence of NAFLD has increased markedly.

As different scholars have reported, the physical and metabolic risk factors and consequences of child malnutrition are not limited to only growth and development; they extend to deficiencies in social skills and improper disease susceptibility (9). The objective of the current review and meta-synthesis is to systematically identify eligible individual studies and pool the magnitude of stunting among under-five children in a different region of Ethiopia, as well as the effect sizes of the determinants of stunting among under-five children in a different region of Ethiopia.

## Materials and methods

### Protocol registration and review reporting

The current review was conducted following the Preferred Reporting Items for Systematic Reviews and Meta-Analyses (PRISMA) guidelines to estimate the magnitude of stunting among children under five years of age in Ethiopia. Before starting the search, a preliminary search for existing reviews (and, ideally, systematic reviews) on the topic was also conducted from the Joanna Briggs Institute (JBI) database of different Reviews and Reports of implementation, the Cochrane Library, CINAHL, PubMed, and EPPI. We also tested the presence of similar ongoing research or reviews related to the current review and confirmed that there was no previous systematic review on the selected topic (http://www.library.UCSF.edu). Finally, the topic was registered at the International Prospective Register of Systematic Review and Meta-analysis (PROSPERO), and proposals were developed to start the search. The registered protocol is found in PROSPERO with registration number CRD42023323568.

## Search strategy

### Study selection and eligibility criteria

The review included articles that were conducted on the incidence, magnitude, and determinants of stunting or chronic undernutrition among underfive children in different regions of Ethiopia. The review participants were only children aged between 0 and 59 months, regardless of their sex, ethnicity, or color. The review included both community- and institution-based studies. The outcome of the review was chronic malnutrition (stunting), its prevalence, and its determining factors. All the study types that were published in the form of original journal articles, conference proceedings, abstracts, master's theses, or a Ph.D. dissertation written in English were included in the systematic review. In addition to the above criteria, all observational studies/community-based cross-sectional studies conducted between 2010 and 2019 were included. Research articles not linked directly to chronic malnutrition, interventional or experimental studies, research with methodological problems, and different types of review articles were excluded. An article that passed the selection criteria was retrieved, and its title, abstract, and full article were assessed for screening.

### Quality assessment and data extraction

To combine the database search results, the software manager for referencing (Endnote V-X7.2) was used, and duplicate data were manually removed. For the meta-analysis, the JBI Meta-Analysis of Statistics Assessment and Review Instrument (MAStARI) was used for the critical appraisal of studies (10). The required data were filtered by two independent reviewers (JM and FN) using a standardized data abstraction format. The data abstraction spreadsheet included the author's name, publication year, country of the research conducted, type of study design applied, total sample size, response rate, and total number of subjects participating by sex. Disagreements or discrepancies between the three reviewers were discussed until a consensus was reached in the presence of another review team member. Furthermore, in the absence of a full-text article, the author of an article communicated through email and another researcher's communication mechanism. If we did not receive any response from the researchers within a month, the document was excluded from the data extraction processes.

### Data analysis and synthesis

Once the data abstraction was completed accordingly, the collected data were categorized, and the sorting was performed using quality control scores; finally, the data were entered into computer software. The data analysis was performed using the command-based windows of STATA Version 14 statistical software. During the analysis, the Cochrane's Q statistic or chi-square test, *I*^2 test,^ and *p* value were used to check for heterogeneity in the outcomes of the interested studies. Again, the heterogeneity was classified based on the *I*^2^ test-related statistical values, such as low if *I*^2^ = 25%, moderate if *I*^2^ = 50%, and high if *I*^2^ is 75% or greater (11), in addition to inspection via a funnel plot. During the interpretation of the results, we found a high heterogeneity score, and we applied a random effects model for further analysis of the Der-Simonian and Laird's pooled effect estimation. The presence of publication bias was assessed by the application of a symmetry funnel plot, and conclusions were generated. Furthermore, publication bias was assessed using Egger's test and Begg's test (12–14). Publication bias was confirmed using a significant *p* value. We performed a sensitivity analysis using a random effects model to assess the influence of a single study on the overall meta-analysis estimate. Furthermore, to identify the source of heterogeneity, a meta-regression was performed using appropriate statistical conclusions, and the presence of a source of heterogeneity was determined.

## Results

The search strategy yielded 743 (using software 699, manual and reference of study search = 42 and 2 recommended from the author) studies, and an additional 331 papers not retrieved in database searches were identified to increase sensitivity. All eligible articles were uploaded into Endnote X^7.2^ software, and 301 duplicates were identified and removed in accordance with Covidence systematic review software (15). All attempts were made to obtain the full texts of the selected articles by searching the web, engaging with the librarian, or contacting the author if necessary. The authors independently screened titles and abstracts for relevance after the major author had been contacted twice two of the papers were sent to the principal investigator by email.

### Search results and characteristics of the studies

**Figure 1:**
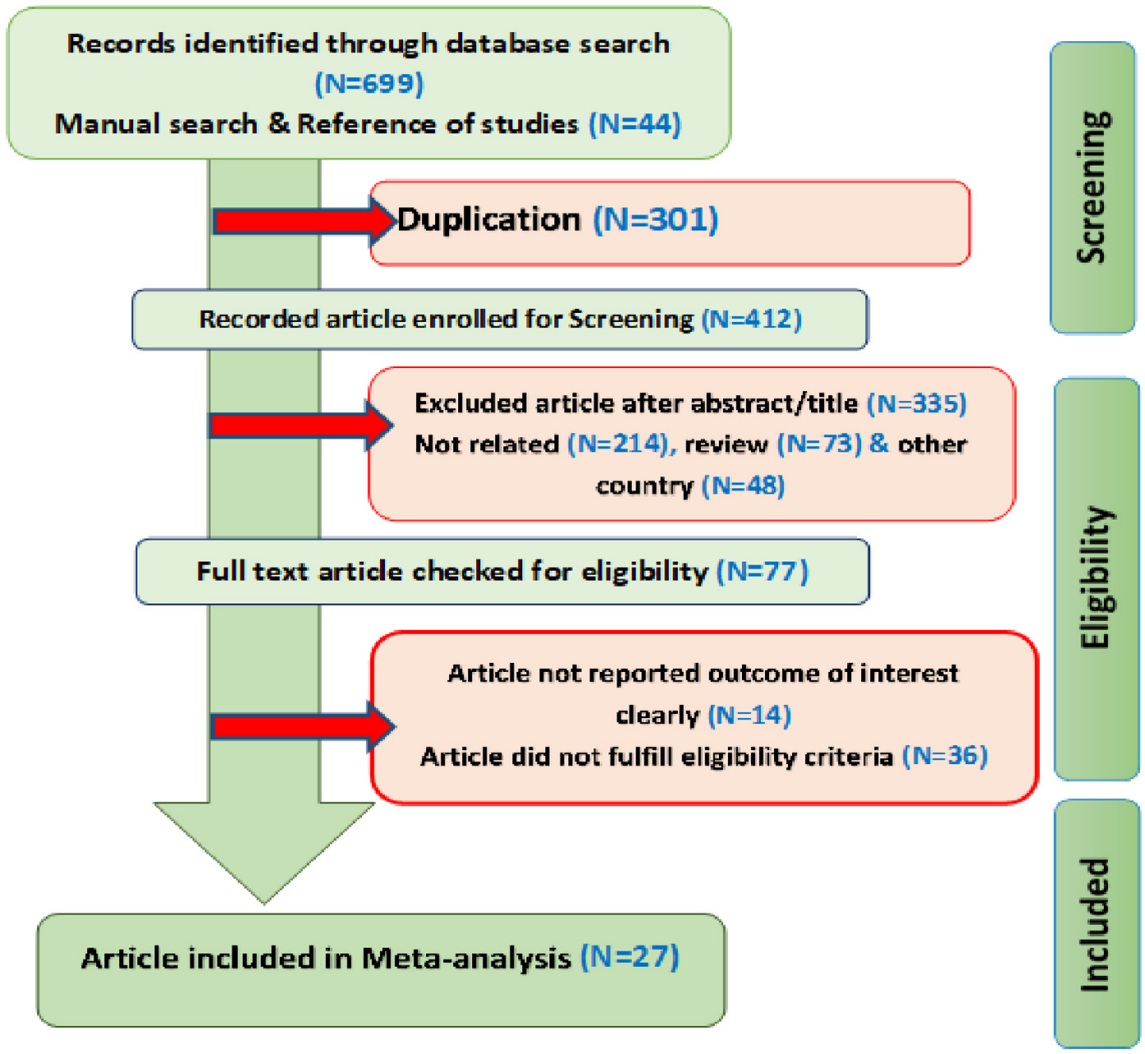
PRISMA flow diagram of included studies in magnitude and determinants of stunting among under-five children in Ethiopia, a systematic review and meta-analysis.

Overall, 27 potentially eligible articles covering 32,448 underfive children were identified (Table 1). Regarding the distribution of individual studies included in this meta-synthesis, ten (10) were from the Amhara region, five (5) were from the SNNP region, four (4) were from Oromia, three (3) were from Somali, two (2) were from the Tigray region, and the remaining two were pooled from EDHS data representing all the regions in Ethiopia. According to the observational descriptive statistics, 7,025 males (42.9%) and 6,189 females (38.5%) were stunted. The PRISMA guidelines-15 were used to describe the article selection process (Figure 1), and all the descriptions of the process are presented in Supplementary Figure S1.

**Table 1:** Data extracted in magnitude and determinants of stunting among under-five children in Ethiopia, a systematic review and meta-analysis.

Pub/N	Author Name	Year Pub/N	Region	Study Design	Sample Size	Age	Subject outcome	Resp/s Rate	No Male outcome	No Female Outcome	Total Male	Total Female
1	Beka Teshome (25)	2009	Amahara	CBCS	622	0–59	269	100	149	120	312	310
2	Gezae Brhane (24)	2014	Tigray	CS	316	0–59	179	100%	102	100	152	164
3	Jemal HAIDAR (26)	2005	Amahara	CBCS	200	6–59	89		39	50	141	59
4	Eskezyiaw Agedew (27)	2015	SNNPR	CBCS	562	6–24	106	99.1	70	36	273	289
5	Beminet Moges (20)	2015	SNNPR	CBCS	734	6–59	260	100	138	22	360	374
6	Beruk Berhanu	2016	SNNPR	CBCS	312	<5 yr	83	86	37	46	157	155
7	Birara Melese Yalew (29)	2014	Amahara	CBCS	844	6–59	399	100	215	184	435	409
8	Selamawit Bekele Geberselassie (34)	2018	Amahara	CBCS	1,287	6–59	636	97.5	297	348	622	665
9	Lamirot Abera (35)	2018	SNNPR	CBCS	398	6–59	166	100	110	56	171	227
10	Misgan Legesse Liben (22)	2016	Afar	CBCS	370	6–59	119	92.3	62	57	178	192
11	Shiferaw Away	2018	Amahara	CBCS	410	6–59	215	97.2	105	110	228	182
12	Sisay Shine (36)	2017	Somali	CBCS	745	6–59	238	96.75	83	155	303	442
13	Amare Tariku (37)	2016	Amahara	CBCS	681	24–59	313	98.4	141	172	365	316
14	Mandefro Asfaw (21)	2015	Oromia	CBCS	778	6–59	307	97.7	206	164	384	394
15	Desalegne Amare (38)	2016	Amhara	CBCS	342	<5 yr	85	100	47	38	168	174
16	Wagaye Fentahun (17)	2016	Amhara	CBCS	633	6–59	365	100	201	164	339	294
17	Zegeye Abebe (39)	2017	Amahara	CBCS	471	24–36	198	98.1	109	89	248	223
18	Zegeye Abebe (39)	2017	Amahara	CBCS	707	6–59	323	92.5	181	142	378	329
19	Solomon Demissie (28)	2013	Somali	CBCS	541	6–59	187	99.4	104	83	263	278
20	Abdibari Ma'alin (40)	2016	Somali	CBCS	694	6–59	232	100	91	141	364	330
21	Zemenu Yohannes Kassa (41)	2017	Oromia	CBCS	384	6–59	147	91	68	79	184	200
22	Tsedeke Wolde (42)	2014	SNNPR	CBCS	358	<5 yr	189	100	89	100	177	181
23	Berihun Megabiaw (43)	2013	Ethiopia	EDHS	9,611	<5 yr	4,065	97.16	2,147	1,923	4,890	4,721
24	Tadiwos Zewdie (16)	2013	Oromia	CS	249	0–59	31	100	17	14	119	130
25	Elema TB (30)	2018	Oromia	CS	217	0–59	117	56.6	61	46	98	119
26	Haile Mekonnen (44)	2019	Ethiopia	EDHS	9,588	0–59	3,644	89	2,031	1,681	4,893	4,695
27	Tesfay Tsegay	2019	Tigray	CS	394	<5 yr	194	98.5	125	69	172	222
	Total				32,448		13,156		7,025	6,189	16,374	16,074

### Overall prevalence of stunting among children under five in Ethiopia

**Figure 2:**
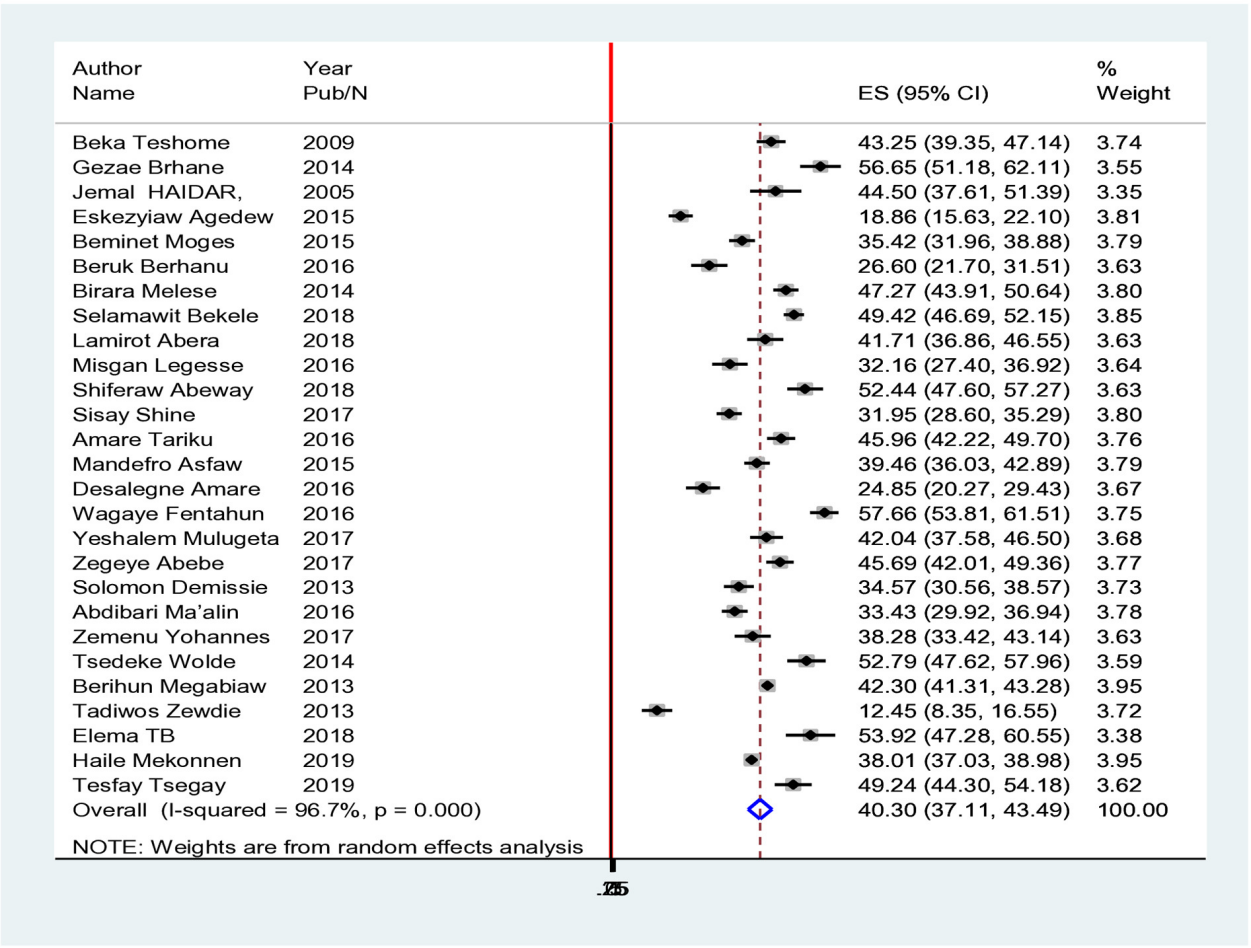
Forest plot of the pooled magnitude and determinants of stunting among under-five children in Ethiopia from 2000–2019. Heterogeneity chi-squared = 364.59 (d.f. = 26) *p* = 0.000. I-squared (variation in RR attributable to heterogeneity) = 92.9%. Estimate of between-study variance Tau-squared = 0.0726. Test of RR = 1: *z* = 2.14, *p* = 0.032.

**Figure 3:**
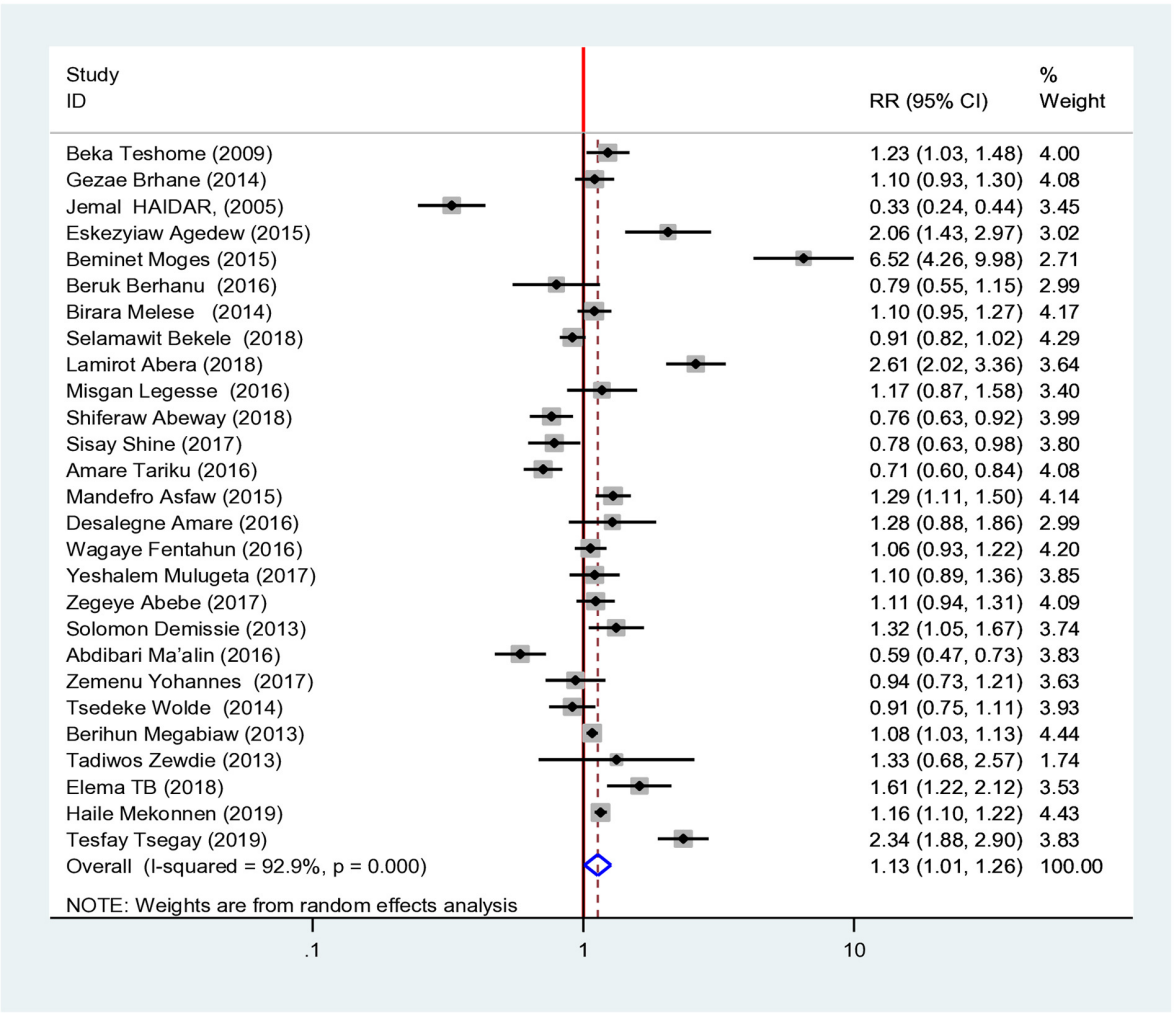
Overall risk ratio systematic review and meta-analysis of magnitude and determinants of stunting among under-five children in Ethiopia, a systematic review and meta-analysis.

The childhood stunting prevalence ranged from Eastern Hararghe (12.45%) the least common (16) to Tigray & Northwest (56.65%) (17), which had the highest prevalence (Figure 2). The overall pooled meta-analysis showed that the incidence of f-stunting was 40.30% (CI: 37.11–43.49 at 95% CI). The pooled effect size of twenty-seven studies showed that male children were 1.13 times more likely to have a risk of stunting than female patients were (RR = 1.13, CI 1.01–1.26 @95%) (Figures 2, 3).

### Publication bias finding/test

The analysis performed to test the variability among the included studies revealed significant heterogeneity in the percentage of total variability in effect measures and Cochran's *Q* statistic. The meta-analysis of 27 individuals potentially included in the study revealed substantial heterogeneity across studies (*I*^2^ =  96.1%, *p* < 0.0010), which suggested that the use of a typical fixed effects model might lead to unreliable approximations because the fixed effects models assume that heterogeneity can be explained by the covariates. Additionally, based on the visual inspection of the forest plot, there was poor overlap of the confidence intervals in each study, which led to type I errors due to unexplained heterogeneity. To avoid this bias, we directly applied a random effects model to estimate the pooled prevalence of child stunting among under-five children in Ethiopia using an inverse variance method (I-V heterogeneity, S1).

### Test for the small study effect

**Figure 4:**
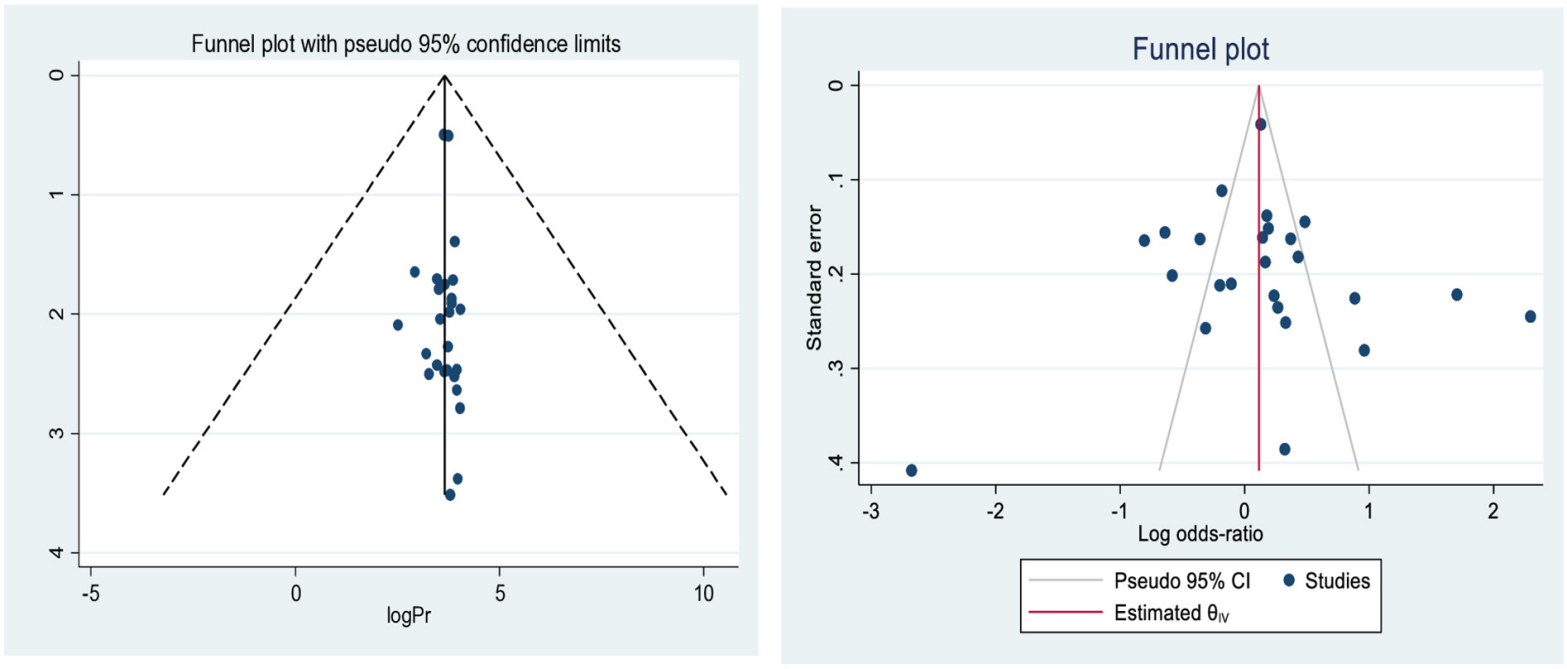
Funnel plot of the pooled magnitude and determinants of stunting among under-five children in Ethiopia from 2000–2019.

The effect of a small study in a systematic review and meta-analysis was reported by statisticians and may indicate publication bias. Where applicable, publication bias was assessed by funnel plot inspection, which might lead to any kind of variation across studies. We investigated the sources of heterogeneity using different statistical techniques for publication bias assessment. The assessments of publication bias were performed using funnel plots and Egger and Begg's statistical tests at the 5% significance level. According to the statistical test, there was no evidence of publication bias. The plotted funnel diagram showed that the bias was nearly distributed symmetrically, and studies even spread evenly over both sides on average, creating a roughly funnel-shaped distribution indicating less publication bias (Figure 4). Both Egger's and Begg's tests indicated no strong evidence for the presence of small-study effects and no statistically significant difference, with a *p*-value of 0.825 according to Egger's test and *p* = 0.381 according to Begg's test for unpublished studies, as shown in the supplementary table below (S2).

### Sensitivity analysis

**Figure 5:**
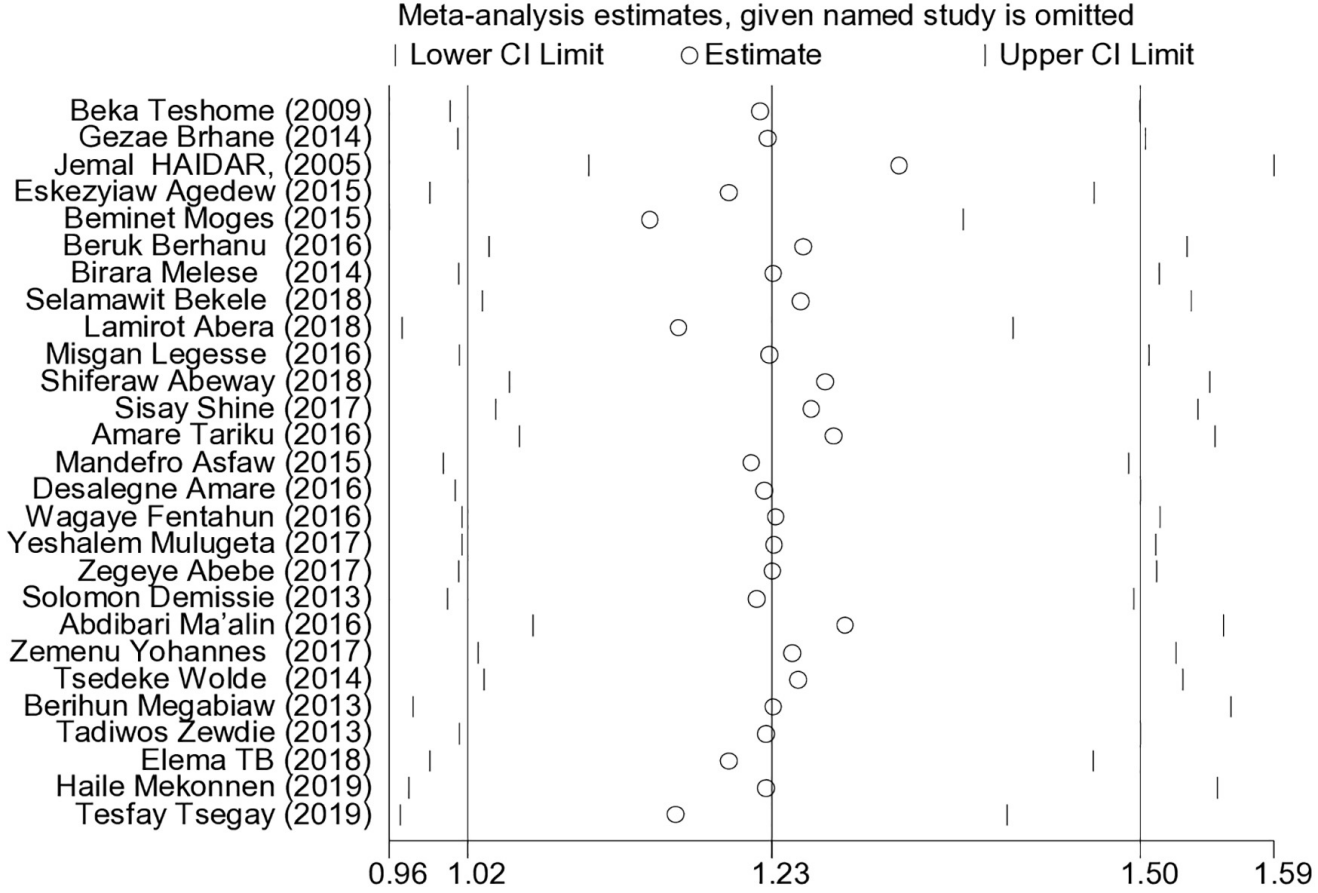
Sensitivity analysis for single study influence on magnitude and determinants of stunting among under-five children in Ethiopia from 2000–2019.

Sensitivity analyses were used to investigate the influence of a single study on the overall meta-analysis estimate using a random effects model, and the results indicated that there was no strong evidence for the influence of a single study (Figure 5).

### Subgroup and meta-regression analysis

Subgroup analyses were performed to reduce prospective random variabilities among eligible studies by considering variables of interest according to the region where the study was conducted. The pooled effect size for the incidence of stunting was highest in the Tigray region (52.567%), followed by the Amhara region (46.28%), and lowest in the SNNP region (31.99%) (Table 2). In Table 3, we used meta-regression analysis to investigate the statistical heterogeneity in the effect estimates, which is an odds ratio in our meta-analysis estimation among the different studies by using the year inthe study was published, the region of the study was included, and the age categories of the child and study design were included as covariates of the eligible articles. Based on our meta-regression, we did not find typical heterogeneity among the included variables.

**Table 2:** Subgroup analysis of the magnitude and determinants of stunting among children under five years old in Ethiopia from 2000–2019.

Study	ES	[95% Conf.]	[Interval]	% Weight
Amahara				
Beka Teshome (25)	43.248	39.354	47.141	2.63
Jemal HAIDAR (26)	44.5	37.613	51.387	0.84
Birara Melese Yalew (29)	47.275	43.907	50.643	3.51
Selamawit Bekele Geberselassie (34)	49.417	46.686	52.149	5.34
Sisay Shine (36)	52.439	47.605	57.273	1.71
Amare Tariku (38)	45.962	42.219	49.705	2.85
Zegeye Abebe (39)	42.038	37.58	46.496	2.01
Zegeye Abebe (39)	45.686	42.014	49.358	2.96
I-V pooled ES	46.744	45.393	48.095	21.84
Tigray				
Gezae Brhane (24)	56.646	51.182	62.109	1.34
Subtotal				
I-V pooled ES	56.646	51.182	62.109	1.34
SNNPR				
Eskezyiaw Agedew (27)	18.861	15.627	22.095	3.81
Beminet Moges (20)	35.422	31.962	38.882	3.33
Beruk Berhanu (23)	26.603	21.699	31.506	1.66
Lamirot Abera (35)	41.709	36.864	46.553	1.7
Tsedeke Wolde (42)	52.793	47.622	57.965	1.49
I-V pooled ES	31.989	30.165	33.812	11.99
Afar				
Misgan Legesse Liben (22)	32.162	27.403	36.922	1.76
I-V pooled ES	32.162	27.403	36.922	1.76
Somali				
Sisay Shine (36)	31.946	28.598	35.294	3.56
Solomon Demissie (28)	34.566	30.558	38.573	2.48
I-V pooled ES	33.023	30.454	35.592	6.04
Oromia				
Mandefro Asfaw (27)	39.46	36.026	42.895	3.38
Zemenu Yohannes Kassa (41)	38.281	33.42	43.143	1.69
Tadiwos Zewdie (16)	12.45	8.349	16.551	2.37
Elema TB (30)	53.917	47.285	60.549	0.91
I-V pooled ES	33.118	30.932	35.303	8.34
Ethiopia				
Berihun Megabiaw (43)	42.295	41.308	43.283	40.87
I-V pooled ES	42.295	41.308	43.283	40.87
Overall				
I-V pooled ES	40.514	39.883	41.145	100

**Table 3:** The meta-regression analysis.

		Random-effects	Meta-regression		Number of obs = 24	
		Method: ML			Residual heterogeneity:	
					tau2 = .1632	
					I2 (%) = 83.21	
					H2 = 5.95	
					R-squared (%) = 75.47	
					Wald chi2 (15) = 52.88	
					Prob > chi2 = 0.0000	
_meta_es	Coefficient	Std. err.	*z*	*P* > z	[95% conf.]	[interval]
YearPubN	0.124865	0.0443125	2.82	0.005	0.0380138	0.211716
Region1	0.203377	0.9179093	0.22	0.825	−1.595692	2.002446
Region2	−0.35074	0.8211173	−0.43	0.669	−1.960105	1.258616
Region3	0.778947	0.8723921	0.89	0.372	−0.9309104	2.488804
Region4	1.753049	0.975862	1.8	0.072	−0.1596058	3.665703
Region5	0.180975	0.7296541	0.25	0.804	−1.24912	1.611071
Region6	1.510633	0.8479281	1.78	0.075	−0.1512757	3.172541
Region7	0.117001	0.8553071	0.14	0.891	−1.55937	1.793372
Region8	−0.83802	0.9054788	−0.93	0.355	−2.612722	0.93669
StudyDesign	0.014677	0.776499	0.02	0.985	−1.507233	1.536587
Age1	0.484001	0.652949	0.74	0.459	−0.7957554	1.763758
Age2	0.359567	0.4948773	0.73	0.467	−0.6103751	1.329508
Age3	−0.32221	0.4788939	−0.67	0.501	−1.260828	0.616401
Age4	−0.533	0.554415	−0.96	0.336	−1.619633	0.553634
Age5	−1.28081	0.3809083	−3.36	0.001	−2.027374	−0.53424
_cons	−251.695	89.37911	−2.82	0.005	−426.8746	−76.5149

Test of residual homogeneity: Q_res = chi2 (8) = 111.13 Prob > Q_res = 0.0000.

## Discussion

However, there are few statistics available regarding the prevalence and contributing factors of stunting in children under five. The pooled magnitude of stunting, as determined by the overall meta-analysis, was 40.30% (CI: 37.11–43.49; 95% CI), greater than the 38.39% seen in the National EDHS data. Fantay (18) and that reported by Aychiluhm SB et al., 2021 (19). The current meta-analysis was consistent with individual-level findings, as the age of the children, male sex, smaller size at birth, economic status of the child (very poor), family categorized under a very-low-income status, poor maternal educational status, and multiple births significantly increased the odds of child stunting, as reported by (18).

The pooled analysis revealed that the major factors associated with underfive stunting were sex, age at birth, maternal educational level and employment status; a family size greater than or equal to five (20); birth child weight; mothers' BMI; diarrhea episode (21); deprivation of colostrum; breastfeeding duration; initiation of early breastfeeding (22); prelacteal feeds (20, 21); food and dietary type; and introduction of complementary feeding time and feeding methods (20, 23–25).

Previously, the undernutrition proportion was reported to be high in female-headed households (26) and high among boys (22), and maternal illness encountered after delivery was identified as a significant independent predictor of childhood stunting (20, 27), which might not be a significant determinant in our current systematic pooling of the meta-analysis. From a different point of view, as reported in the current meta-analysis and a report by Fantay Gebru et al. (18), at the community level, the household water risk of being stunted was 0.49 times lower than that of using unsafe water (28), which was again not a significant predictor in our meta-analysis. The reason might be the exclusion of outcome criteria among those articles. In another Ethiopian study by Biruk Birhanu in 2016, children older than 24 months were four times more likely (AOR = 3.97) to be stunted than were those younger than 12 months old; these findings were not significant in the present meta-analysis and are different from previous findings (18). However, similar to the findings of Yalew, taking honey in the morning to the child was significantly and independently associated with being underweight (29). Additionally, there is a significant report regarding the supplementation of vitamin A, as those who did not complete vitamin A supplementation were more significantly (AOR = 1.298) malnourished than children who completed vitamin A supplementation in the last six months, as reported by Elema et al. (30).

## Conclusion

As reported by different Ethiopian scholars (18, 29, 30) in the current meta-synthesis, stunting among under-five children was found to be significantly associated with child weight at birth, age of the child, mothers' BMI, diarrhea episodes, breastfeeding durations, deprivation of colostrum, and early initiation of breastfeeding, where the child's age significantly increases the risk of being stunted. This can be improved by taking into account Ethiopian families and the reasons why they account for a small portion of the country's female infant population, particularly when it comes to gender issues. The primary conclusion of this meta-analysis, which (18) supports, is that the difficulties related to childhood gender stunting can be resolved if mothers' and girls' education can be viewed as a public health strategy or a substitution mechanism.

The other issues include differences in food types and living environments, as reported by (31); sociodemographic/socioeconomic and cultural differences (32); and the possibility that geographical location might influence the nutritional status of children, which was again clearly observed in many articles during the systematic review. These findings are again supported by findings from Ethiopians, Indians, Ghanaians, and the Democratic Republic of the Congo (DRC), which might need an interventional investigation (25, 30, 33).

Therefore, critical evaluation of existing health facilities to promote dietary diversity, sex, maternal health education, maternal nutritional status (counseling of nutritional practices), increasing nutritional literacy, early initiation of breastfeeding, control of diarrhea, and improved health delivery, in general, could be recommended to address intergenerational challenges. Child stunting is not only family-focused; it can be a combination of multidisciplinary contributions, as multiple systems can impact the health status of children. In Ethiopia, traditional food attitudes and Indigenous food diversity practices to increase diet diversity, nitrification, and fortification, diet biodiversification, enrichment, and bioavailability may change the trends of national stunting as a means of urgent solution.

## Data Availability

The original contributions presented in the study are included in the article/Supplementary Material, further inquiries can be directed to the corresponding author.
