# Supplementary figures and images for: Magnitude and determinants of stunting among children under five years of age in Ethiopia: a systematic review and meta-analysis
Source: Front Pediatr. 2025 Jun 9;13:1499921. doi: 10.3389/fped.2025.1499921 (PMC12183166; doi:10.3389/fped.2025.1499921)

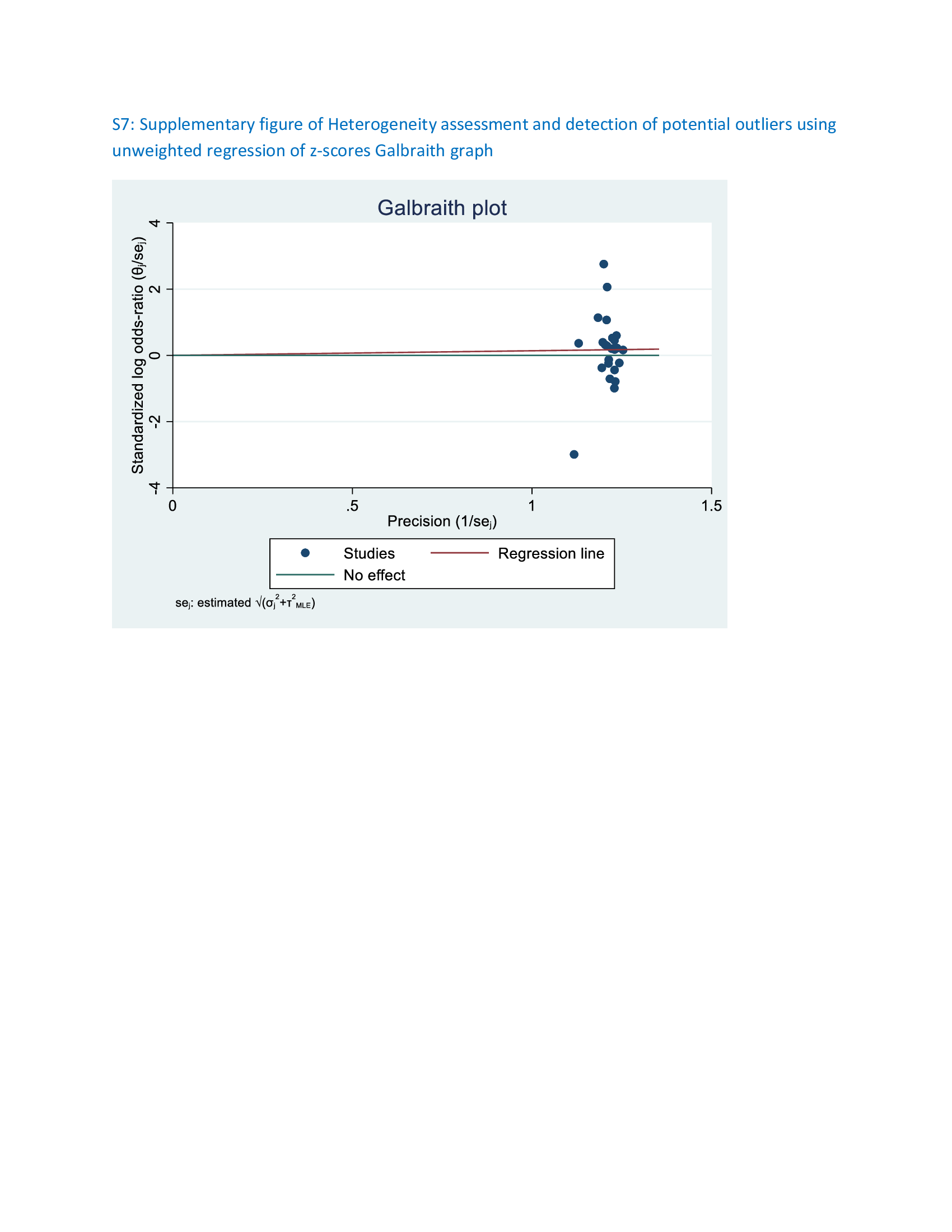

Supplement: Supplementary file 1 [file Datasheet1.zip › S7_fig.pdf.tiff]

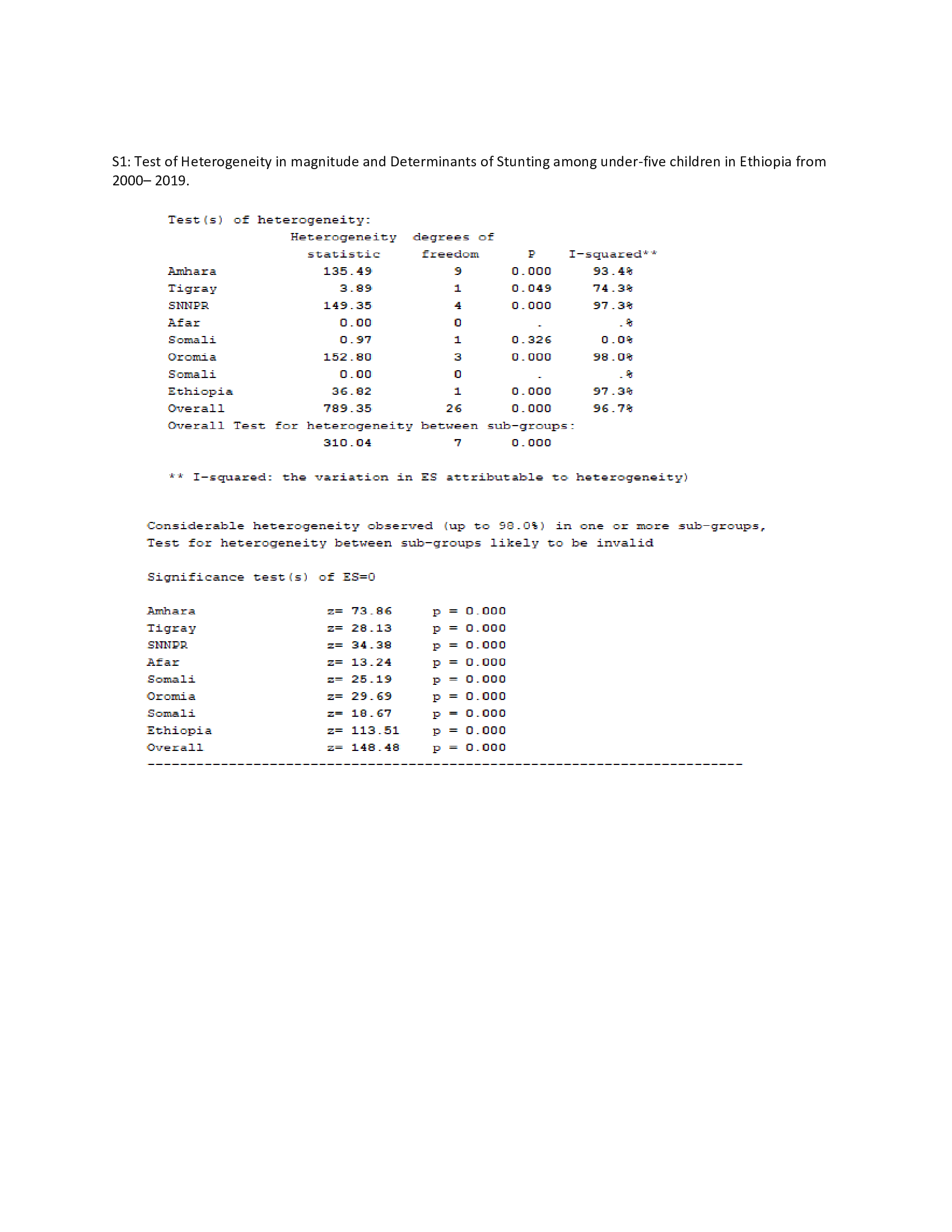

Supplement: Supplementary file 1 [file Datasheet1.zip › S1_Fig.pdf.tiff]

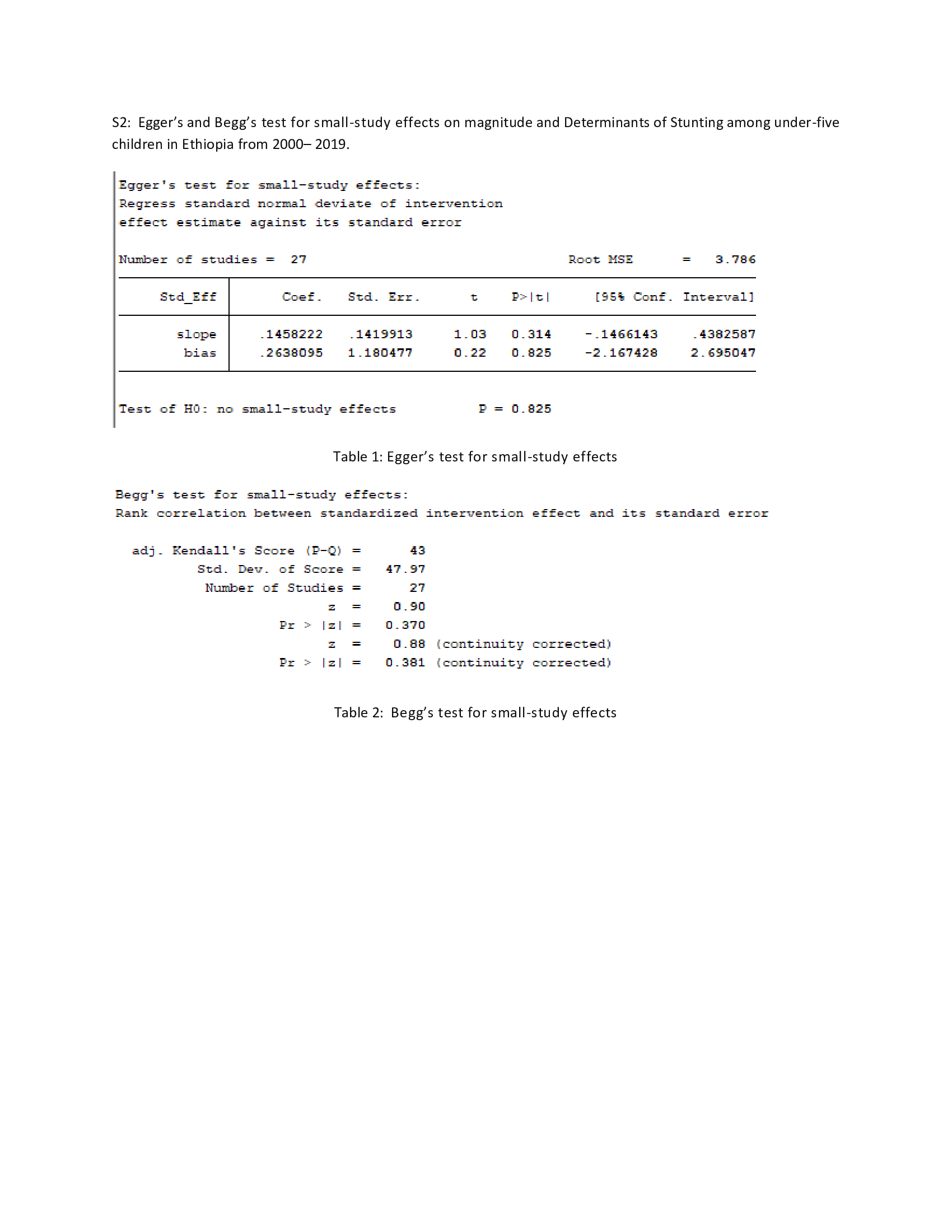

Supplement: Supplementary file 1 [file Datasheet1.zip › S2_fig.pdf.tiff]

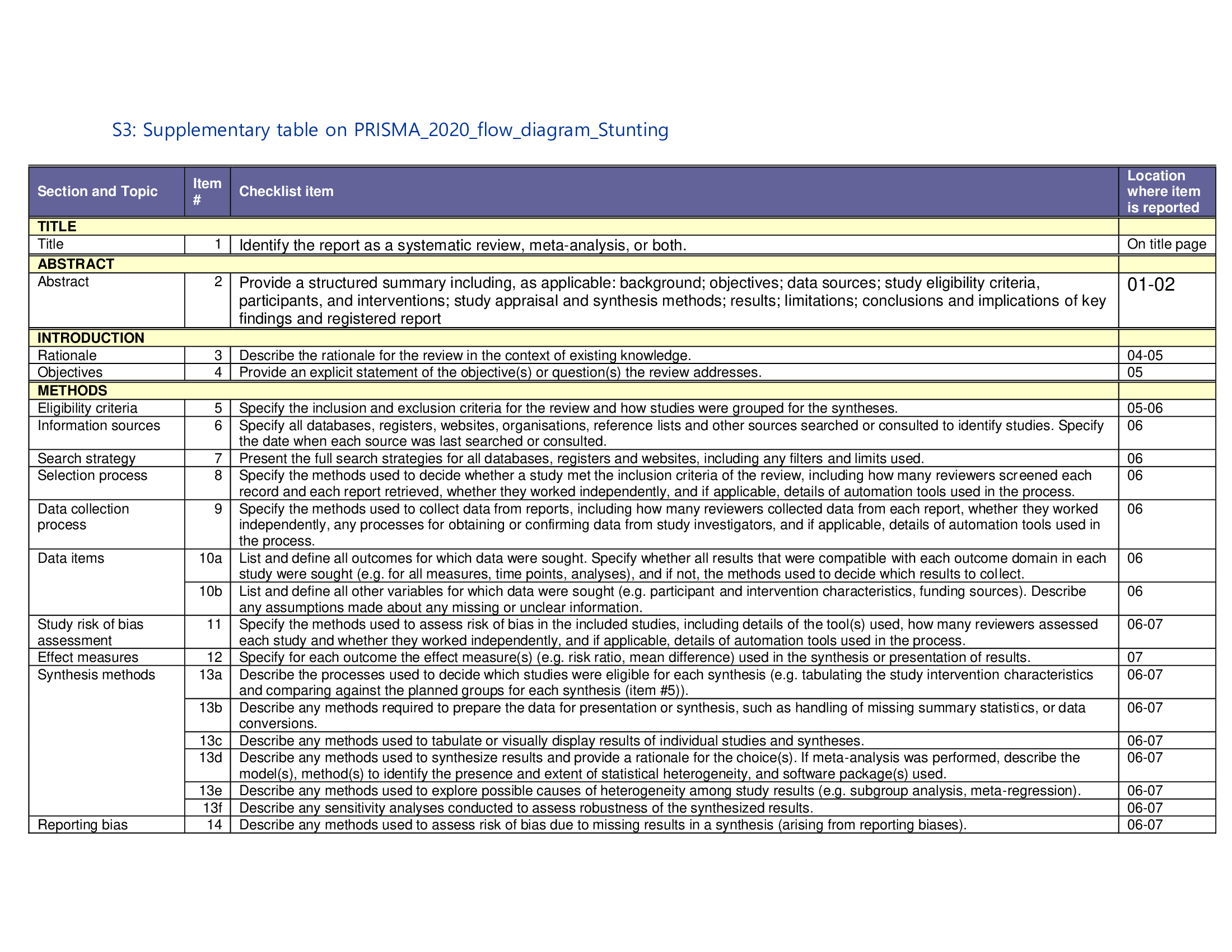

Supplement: Supplementary file 1 [file Datasheet1.zip › S3_fig.pdf.tiff]

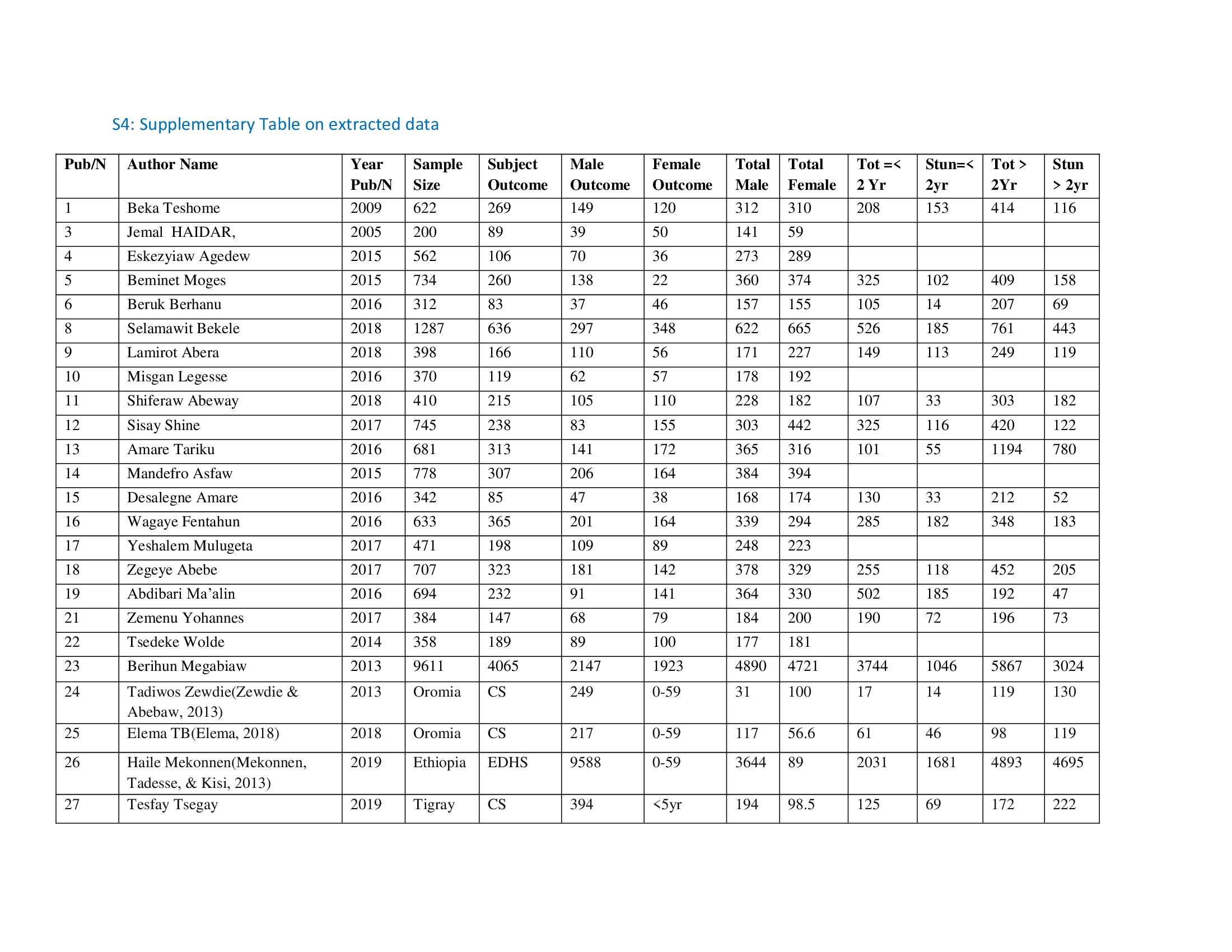

Supplement: Supplementary file 1 [file Datasheet1.zip › S4_fig.pdf.tiff]

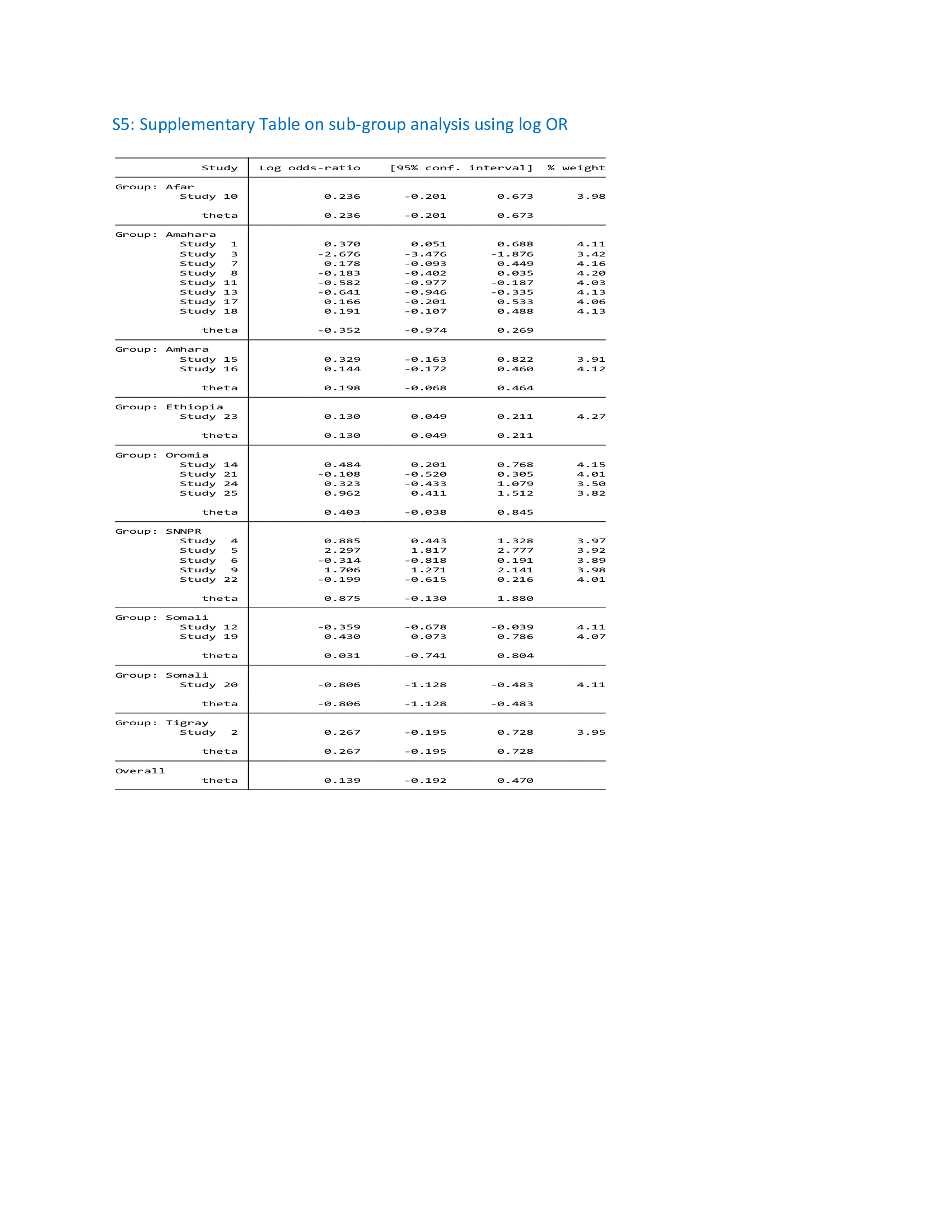

Supplement: Supplementary file 1 [file Datasheet1.zip › S5_fig.pdf.tiff]

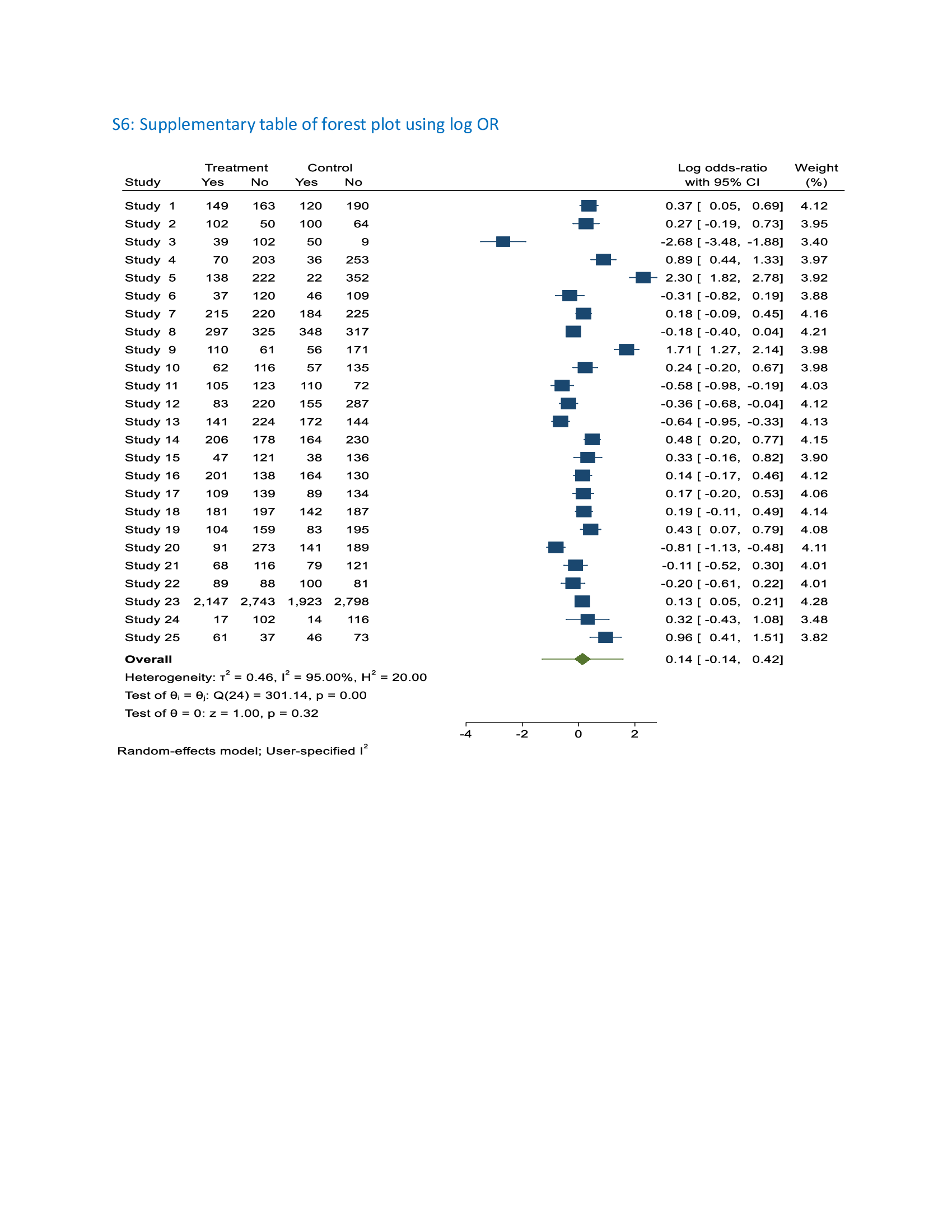

Supplement: Supplementary file 1 [file Datasheet1.zip › S6_fig.pdf.tiff]
